# Household Food Insecurity Alters Gut Microbiome Composition and Enriches *Sutterella* in Ethiopian Schoolchildren

**DOI:** 10.3390/nu18040680

**Published:** 2026-02-20

**Authors:** Angie Zhu, Fisseha Bonja Geleto, Musa Mohammed Ali, Hagos Ashenafi, Berhanu Erko, Bineyam Taye

**Affiliations:** 1 Department of Mathematics, Colgate University, Hamilton, NY 13346, USA; azhu@colgate.edu; 2 Department of Biology, Colgate University, 13 Oak Dr., Hamilton, NY 13346, USA; 3 School of Medical Laboratory Science, College of Medicine and Health Sciences, Hawassa University, Hawassa 1235, Ethiopia; fisseha.bonja@aau.edu.et (F.B.G.); ysnmss@yahoo.com (M.M.A.); 4 Aklilu Lemma Institute of Health Research, Addis Ababa University, Addis Ababa 1000, Ethiopia; hagosashenafi@aau.edu.et (H.A.); berhanue@yahoo.com (B.E.); 5 Global Public Environmental Health, Colgate University, 13 Oak Dr., Hamilton, NY 13346, USA

**Keywords:** food insecurity, gut microbiome, *Sutterella*, 16S rRNA sequencing, school children, Ethiopia

## Abstract

**Background:** Household food insecurity (HFI) adversely affects child development by restricting caloric intake, dietary diversity, and food quality. Since diet is a key factor influencing the gut microbiome, HFI may negatively impact health by altering microbial communities. However, direct evidence linking HFI to changes in the gut microbiome is limited. Therefore, we investigated the effects of HFI as a composite variable and used individual HFI assessment questions as specific proxies for dietary deprivation on the gut microbiome in a group of Ethiopian schoolchildren. **Methods:** Fecal samples were collected from 57 school-aged children in Ethiopia, and microbial profiles were established using 16S rRNA amplicon paired-end sequencing. Food insecurity was assessed using the Household Food Insecurity Access Scale (HFIAS). **Results:** We observed no significant differences in alpha diversity across food security status (Wilcoxon *p* > 0.05). However, beta diversity analysis revealed a significant shift in microbiome composition between food-secure and food-insecure individuals (Bray–Curtis dissimilarity; PERMANOVA, *p* < 0.05). Further analyses of individual HFIAS questions as specific proxies for dietary deprivation showed that limited dietary variety, consumption of disliked foods, and reduced meal size were each associated with significant changes in microbial compositions (PERMANOVA; all *q* < 0.05). Differential abundance analyses consistently identified *Sutterella* as significantly more abundant among food-insecure participants (composite model *q* = 0.11; component-specific models *q* < 0.05). Additionally, a microbial feature-based machine learning model accurately predicted food security status (AUC = 0.81), with *Sutterella* emerging as the top predictive feature. **Conclusions:** Our findings suggest that food insecurity metrics are associated with alterations in gut microbial composition. The consistent enrichment of *Sutterella* in food-insecure children in this study suggests the need for future mechanistic studies to explore its role in mediating the effects of food insecurity.

## 1. Introduction

Food insecurity (FI) is defined as limited or uncertain access to nutritionally adequate and safe foods or limited ability to acquire acceptable foods through socially accepted means [1]. According to the United Nations (UN) State of Food Security and Nutrition in the World (SOFI), approximately 2.3 billion people, nearly 30% of the global population, faced food insecurity in 2021 [2]. Nowhere is this crisis more severe than in Africa, where 61% of the population faces moderate or severe food insecurity, attributed to climate shocks, conflict, and economic slowdowns [3]. Researchers who studied the consequences of food insecurity reported a wide array of adverse health outcomes, including obesity [4,5], type 2 diabetes [6,7,8], cardiovascular disease [9,10], poor mental health outcomes [11,12], and undernutrition [13,14]. Despite these well-documented associations, the impact of food insecurity on the gut microbiome remains unexplored. The gut microbiome is integral to metabolic functions, including digestion, energy harvest, and vitamin synthesis [15,16,17,18], and its composition is susceptible to dietary changes [19,20]. While extensive research has explored the effects of specific diets, such as the beneficial role of the Mediterranean diet [21,22] or the detrimental impact of highly processed foods [23,24], the influence of broader socioeconomic determinants of diet, as measured by the food insecurity scale, remains less clear. Furthermore, existing microbiome studies on nutrient deprivation have primarily focused on clinical malnutrition [25,26] or controlled caloric restriction [27], overlooking the complex psychosocial and economic realities of household food insecurity.

Household food insecurity is a complex, multidimensional problem that extends beyond poor diet quality, including psychological anxiety about food, insufficient quantity, and socially driven reductions in food intake [27]. This complexity suggests that its impact on the gut microbiome may be through pathways distinct from nutrition alone. To date, only a few studies have looked at the impact of food insecurity on the gut microbiome. Mohr et al. [28] investigated the relationship between food security status and the gut microbiome among United States (US) college students, showing that food-insecure students had higher microbial diversity and greater abundance of *Enterobacteriaceae* and *Eisenbergiella* than food-secure counterparts. A similar increase in abundance of *Eisenbergiella* and/or *Eubacterium* among food-insecure individuals was reported by Eggers et al. [29], using the adult population from Wisconsin, USA. However, another study using infants aged 2 to 6 months found a lower abundance of *Veillonella* spp. in infants born to food-insecure mothers than in those born to food-secure mothers [30]. While these studies indicate that food insecurity can be associated with an altered gut microbiome, their findings are based solely on populations in high-income settings. The context of food insecurity in a resource-rich environment, often characterized by the consumption of inexpensive, energy-dense but nutrient-poor foods [31], is fundamentally different from that in low-income countries, where food insecurity is more directly linked to chronic undernutrition, micronutrient deficiencies, and a lack of dietary diversity [32,33,34]. It is therefore unclear whether the microbial signatures of food insecurity identified in U.S. populations, such as increased *Eisenbergiella*, are applicable to contexts like Ethiopia, where the stress of food scarcity is compounded by its more severe nutritional consequences. We therefore leverage our data from Ethiopian school children to (1) examine the differences in gut microbiome composition between food-secure and food-insecure children, (2) investigate how specific domains of food insecurity measurement impact the gut microbiome, and (3) apply machine learning on gut microbiome data to predict food security status and identify potential microbial biomarkers.

## 2. Methods

### 2.1. Study Design

This cross-sectional study was conducted in Hawela Tula, Hawassa, Sidama Regional State, Ethiopia. Participants were recruited from primary school children attending full-cycle primary schools in the Hawassa city administration during the 2023 academic year. Eligible children aged 5–18 years were randomly selected. The eligibility criteria included no terminal illness, no other diseases, and had not received anti-helminthic treatment or iron supplementation in the past three months. Further requirements included residence in the area for at least six months and availability during the study period. Children with guardians who were seriously ill and unable to provide informed consent were excluded.

### 2.2. Food Security Status

Food security status was assessed through the Household Food Insecurity Access Scale (HFIAS) [35]. Study participants were asked to recall the past 30 days and were interviewed based on the nine standard HFIAS questions. The questions were the following:

(1) Did you worry that your household would not have enough food? (2) Were you or any household member not able to eat the kinds of foods you preferred because of a lack of resources? (3) Did you or any household member have to eat a limited variety of foods due to a lack of resources? (4) Did you or any household member have to eat some disliked foods because of a lack of resources? (5) Did you or any household member have to eat a smaller meal because there was not enough food? (6) Did you or any household member have to eat fewer meals in a day because there was not enough food? (7) Was there ever no food to eat of any kind in your household because of lack of resources? (8) Did you or any household member go to sleep at night hungry because there was not enough food? (9) Did you or any household member go a whole day and night without eating anything because there was not enough food?

Based on the responses “never”, “rarely” (once or twice in the past 4 weeks), “sometimes” (3 to 10 times in the past 4 weeks), and “often” (more than 10 times in the past 4 weeks), a score from 0 to 3 was assigned, respectively. The scores for each question were summed to calculate a total score for each participant. A total score of 0–1 was classified as food-secure, while a score of 2–27 was classified as food-insecure.

### 2.3. Sample Collection and DNA Extraction

A detailed specimen collection procedure was described previously [36]. Briefly, fecal samples were collected in leak-proof plastic containers and divided into two portions. The first portion was used for a parasitological examination and was processed immediately for microscopic analysis of helminths using direct wet mounts and the Kato–Katz technique at Hawassa University College of Medicine and Health Sciences, School of Medical Laboratory Science Microbiology Laboratory. The second portion was collected and stored in Norgen Stool Nucleic Acid Collection and Preservation Tubes (Norgen Biotek Corp, Thorold, ON, Canada). This portion was transported to Colgate University in the USA and stored at −80 °C. DNA extraction was performed using the DNeasy PowerSoil Pro kit (Qiagen, Hilden, Germany) according to the manufacturer’s instructions, utilizing up to 250 mg of fecal material per extraction. After extraction, aliquoted DNA samples were shipped to the MR DNA molecular laboratory in Shallowater, TX, USA, for sequencing on the Illumina MiSeq platform, following standard Illumina protocols. The raw sequence data were provided in FASTQ file format and were demultiplexed using FASTQ Splitter 64-bit v19.07.10.

### 2.4. Raw Sequence Processing

Raw 16S rRNA gene sequences were processed using the Nephele platform (https://nephele.niaid.nih.gov) [37]. Briefly, primer sequences were trimmed, and reads were quality-filtered using Trimmomatic. Subsequently, sequences were denoised, merged, and checked for chimeras using DADA2 to generate a table of amplicon sequence variants (ASVs), which were then taxonomically classified against the SILVA database. Following this, a phylogenetic tree was constructed, and all downstream statistical analyses and visualizations were performed in R using the phyloseq data object (v1.46.0) [38].

### 2.5. Statistical Data Analysis

All analyses were performed in R (v4.5.1). First, alpha diversity was calculated to assess microbial diversity among samples. Chao1 [39] and Shannon [40] indices were calculated via the estimate_richness() function from the phyloseq package (v1.52.0) [38]. Statistical differences across groups were calculated using the Wilcoxon test. Boxplots were rendered with ggplot2 (v3.5.2) [41]. Following this, beta diversity (between-sample diversity) was measured using the Bray–Curtis dissimilarity index [42]. These distances were calculated using the phyloseq::distance() function. Principal coordinate analysis (PCoA) was then performed and visualized with a dotted ellipse representing a 95% confidence interval through MicrobiotaProcess (v1.1.13) [43]. Permutational multivariate analysis of variance (PERMANOVA) was conducted to test for statistically significant differences between groups via the adonis2() function from the vegan package (v2.7.1) [44] with default settings. Genus-level microbial differences were visualized using ggplot2. Relative abundance levels were calculated at the genus level, and the top 15 most abundant genera were shown. Linear discriminant analysis effect size (LEfSe) was performed through run_lefse() via microbiomeMarker (v1.13.2) [44]. Genus-level features were removed, and default parameters of 0.01 and 3 were used for the Wilcoxon cutoff and LDA score, respectively. Features were annotated based on their taxonomic level. Differential abundance analysis at the genus-level was performed through Maaslin2 (v1.22.0) [45]. Relative abundances were normalized using total sum scaling (TSS), and the multivariate model was adjusted for age and sex. The model was further adjusted for multiple testing through the Benjamini–Hochberg procedure [46]. Volcano plots were generated to visualize effect sizes and significance, with *q* < 0.2 as the cutoff. Genus-level boxplots were created to visualize relative abundance differences for significantly associated genera.

### 2.6. Machine Learning

To assess whether gut microbiome profiles could predict household food security status, we implemented a machine learning model using an XGBoost classifier [47] and 10-fold cross-validation. Genus- and family-level abundances were aggregated, and features present in less than 5% of samples were filtered. Abundance counts were transformed with centered log-ratio, and features with near-zero variance across samples were removed. The 10-fold cross-validated XGBoost model was trained using caret (v7.0.1) [48]. Model hyperparameters were tuned using grid search, and the final model was selected based on the highest cross-validated classification accuracy.

To assess the model’s discriminative ability between food-secure and food-insecure groups, receiver operating characteristic (ROC) analysis was conducted using predicted probabilities for the positive class (food-secure) by the XGBoost model. The ROC curve and area under the curve were computed through pROC (v1.18.5) [49] and visualized with ggplot2. To identify the most informative features, the top 10 most important features were extracted from the trained model and visualized as a horizontal bar plot. Features were annotated based on their taxonomic level. To visualize abundance patterns across study participants, a heatmap of the top 10 most important features was created using CLR-transformed abundance data via pheatmap (v1.0.13) [50]. Samples were annotated and grouped by food security status, and each feature (row) was scaled by z-score.

### 2.7. Data and Code Availability

The full pipeline for data analysis and machine learning described in “Methods” is available at the GitHub repository: https://github.com/azh-a/HFI_gutMicrobiome (accessed on 16 February 2026).

### 2.8. Ethics

Ethical clearance was obtained from the Institutional Research Ethics Review Committee of the Aklilu Lemma Institute of Health Research, Addis Ababa University (Ref.no: ALIPBIRERC/91/2015/22). Permission was also secured from the Health and Education Department of the sub-city for each selected school. We obtained written or fingerprint consent from parents or legal guardians after informing them of the study procedures and aims. Assent was also obtained from the children. Confidential numerical identifiers were assigned to each child to ensure participant privacy, and all participant information remains password-protected in electronic files. Children were also informed of their ability to withdraw from this study at any time without jeopardizing their right to receive services at the school.

## 3. Results

### 3.1. Study Population Characteristics

Table 1 presents the characteristics and demographic information of our study cohort stratified by food security status. In our study of 57 Ethiopian children, 63% of participants were female, and the majority (93%) resided in rural residences. The mean age of the children was 10.8 years (SD 2.2 years), ranging from 7 to 16 years. The average body mass index (BMI) was underweight, at 17.1. In total, 56% of participants were identified as food-secure through the Household Food Insecurity Access Scale (HFIAS) questionnaire. No associations between various sociodemographic factors and food security status were detected.

### 3.2. Gut Microbiome Composition Is Impacted by Food Security Status

**Figure 1:**
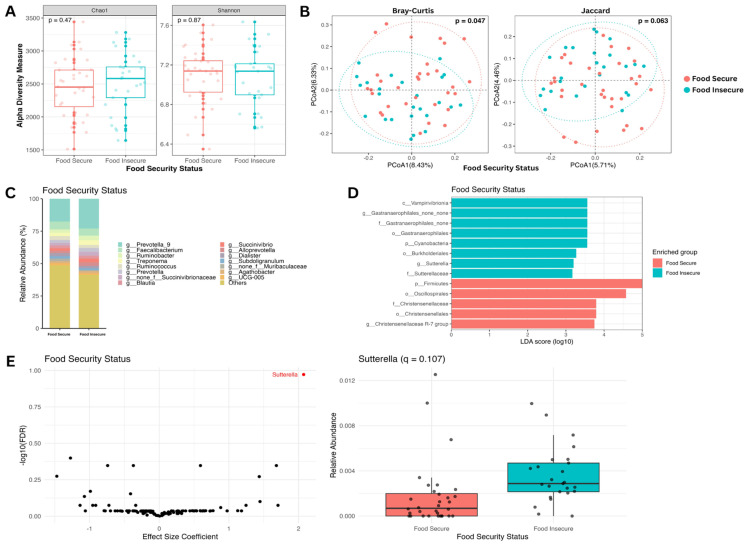
Taxonomic analysis of the gut microbiome by food security status (food-secure: 32 (56%) vs. food-insecure: 25 (44%)). (**A**) Alpha diversity boxplots showing Chao1 (**left**) and Shannon (**right**) indices. Differences in the means of the alpha diversity metrics between the food-secure (red) and food-insecure (blue) groups are not significant by the Wilcoxon test. (**B**) Beta diversity PCoA plots showing Bray–Curtis (**left**) and Jaccard (**right**) metrics. Using PERMANOVA, the difference between the beta diversity metric clustering of the two groups is significant (Bray–Curtis, *p* < 0.05). (**C**) Relative abundance bar plot showing the top 15 most abundant genera comparing food-secure and food-insecure groups. (**D**) Linear discriminant analysis effect size bar plot. Bars represent taxa with significant group abundance differences (Wilcoxon *p* < 0.01; LDA score > 3). (**E**) Genus-level differential abundance analysis using (after adjusting for age, sex, and multiple testing) a volcano plot (**left**) and a relative abundance boxplot (**right**). The differential abundance of *Sutterella* suggested enrichment in the food-insecure group; however, this did not reach statistical significance after multiple-testing correction (*q* = 0.11).

To explore how food security status influences gut microbiome composition, we first investigated alpha and beta diversity. Alpha diversity indices measuring species richness and evenness among samples (Chao1 and Shannon) were not significantly different by food security status (Wilcoxon *p* > 0.05; Figure 1A). However, beta diversity metrics representing the dissimilarity between the abundance and presence of taxa between samples showed moderate significance between the two study groups (Bray–Curtis, PERMANOVA, *p* < 0.05). Principal coordinate analysis (PCoA) revealed clear compositional differences between food-secure and food-insecure individuals (Figure 1B). To characterize specific microbial differences further, we compared the relative abundances of key taxa between the two study groups. While patterns remain similar, the relative abundance of *Prevotella 9* appeared to be greater in the food-insecure group compared to the food-secure group (Figure 1C). Linear discriminant analysis effect size (LEfSe) identified five taxa significantly enriched in the food-secure group and eight taxa enriched in the food-insecure group (Wilcoxon *p <* 0.01 and LDA score *>* 3; Figure 1D). Notably, the family *Christensenellaceae* was enriched in the food-secure group, while the genus *Sutterella* was enriched in the food-insecure group (Figure 1D). Differential abundance analysis at the genus level showed a trend toward enrichment of *Sutterella* in the food-insecure group (*q* = 0.11) after adjusting for multiple testing, age, and sex (Figure 1E). Relative abundance plots of *Sutterella* across food security status confirmed higher *Sutterella* abundance in food-insecure individuals (Figure 1E).

### 3.3. Gut Microbiome Composition Is Impacted by Food Variety Consumption

Food security status is a composite variable constructed using the nine standard questions indicated by the HFIAS questionnaire. To better understand which components of food insecurity shape gut microbiome composition, we investigated each individual question of the HFIAS questionnaire separately. We next highlight the questions most linked to microbial variation.

**Figure 2:**
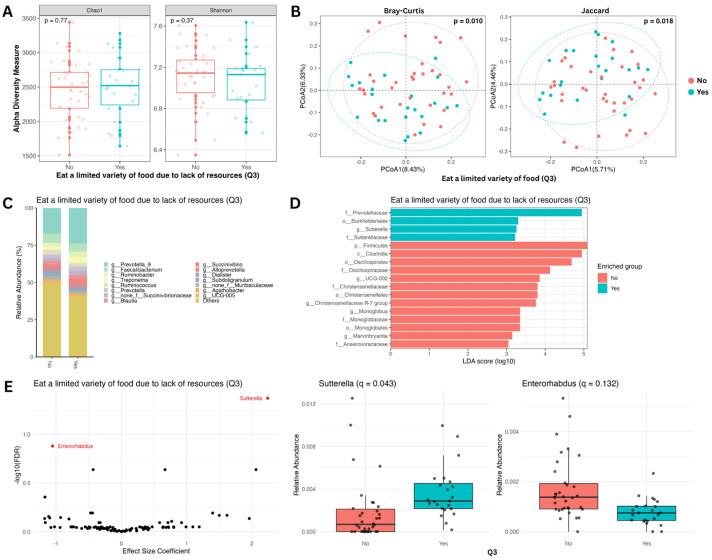
Taxonomic analysis of gut microbiome by question 3 of the HFIAS questionnaire: Did you or any household member have to eat a limited variety of foods due to a lack of resources (yes: 18 (32%); no: 39 (68%))? (**A**) Alpha diversity boxplots showing Chao1 (**left**) and Shannon (**right**) indices. Differences in the means of the alpha diversity metrics between yes (blue) and no (red) groups are not significant by the Wilcoxon test. (**B**) Beta diversity PCoA plots showing Bray–Curtis (**left**) and Jaccard (**right**) metrics. Using PERMANOVA, the difference between the beta diversity metric clustering of the two groups is significant (Bray–Curtis *p* < 0.05 and Jaccard *p* < 0.05). (C) Relative abundance bar plot showing the top 15 most abundant genera comparing yes and no groups. (**D**) Linear discriminant analysis effect size bar plot. Bars represent taxa with significant group abundance differences (Wilcoxon *p* < 0.01; LDA score > 3). (**E**) Genus-level differential abundance analysis (after adjusting for age, sex, and multiple testing) volcano plot (**left**) and relative abundance boxplots (**right**). The differential abundance of *Sutterella* was significant (*q* < 0.05), while that of *Enterorhabdus* showed a trend toward differential abundance but did not reach statistical significance after multiple-testing correction (*q* < 0.15).

The question “*Did you or any household member have to eat a limited variety of foods due to a lack of resources?*” was particularly informative. Households that reported a limited variety of foods due to resource constraints exhibited a distinct gut microbiome composition compared to those that did not report such constraints. While no significant differences were observed in alpha diversity (Figure 2A), beta diversity analysis revealed a significant separation between the groups (PERMANOVA, Bray–Curtis, and Jaccard: *p* < 0.02; Figure 2B). To identify taxa driving this separation, we performed differential abundance analysis. We found four taxa, including *Prevotella 9* (Figure 2C), enriched in the group experiencing food variety limitations and 13 taxa enriched in the control group (Wilcoxon *p* < 0.01 and LDA score > 3; Figure 2D). Furthermore, differential abundance analysis using a conservative model confirmed the significant enrichment of *Sutterella* (*q* < 0.05) in the “limited variety” group while *Enterorhabdus* showed suggestive enrichment in the “no” response individuals, though this association did not survive multiple-testing correction (*q* < 0.15; Figure 2E).

### 3.4. Gut Microbiome Composition Is Impacted by Disliked Food Consumption

**Figure 3:**
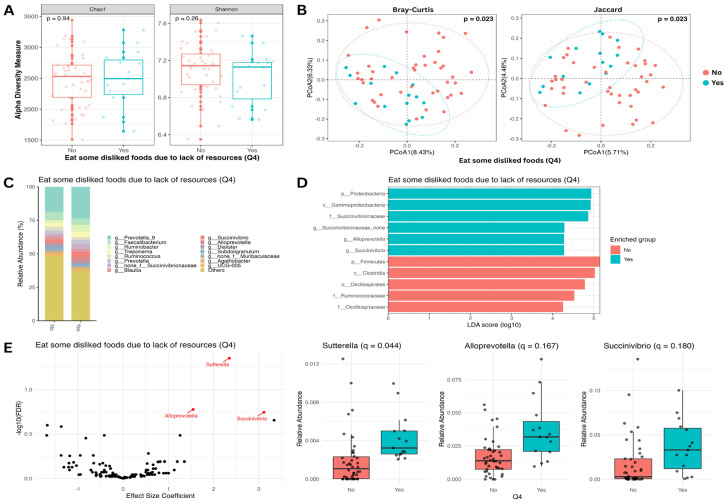
Taxonomic analysis of gut microbiome by question 4 of the HFIAS questionnaire: Did you or any household member have to eat some disliked foods because of a lack of resources (yes: 15 (26%); no: 42 (74%))? (**A**) Alpha diversity boxplots showing Chao1 (**left**) and Shannon (**right**) indices. Differences in the means of the alpha diversity metrics between yes (blue) and no (red) groups are not significant by the Wilcoxon test. (**B**) Beta diversity PCoA plots showing Bray–Curtis (**left**) and Jaccard (**right**) metrics. Using PERMANOVA, the difference between the beta diversity metric clustering of the two groups is significant (Bray–Curtis *p* < 0.05 and Jaccard *p* < 0.05). (**C**) Relative abundance bar plot showing the top 15 most abundant genera comparing yes and no groups. (**D**) Linear discriminant analysis effect size bar plot. Bars represent taxa with significant group abundance differences (Wilcoxon *p* < 0.01; LDA score > 4). (**E**) Genus-level differential abundance analysis (after adjusting for age, sex, and multiple testing) volcano plot (**left**) and relative abundance boxplots (**right**). The differential abundance of *Sutterella* was statistically significant after multiple-testing correction (*q* < 0.05). In contrast, *Alloprevotella* and *Succinivibrio* showed suggestive differences between groups but did not reach the predefined threshold for statistical significance (*q* < 0.20).

We next investigated whether the consumption of disliked foods due to resource constraints, another dimension of food insecurity, was associated with variations in the gut microbiome. Similarly to the findings on food variety, no significant differences in alpha diversity were observed between groups (Figure 3A). However, beta diversity analysis revealed a significant separation of microbial community structures (PERMANOVA, *p* < 0.05; Figure 3B). Linear discriminant analysis (LEfSe) identified six taxa enriched in individuals who consumed disliked foods and five taxa enriched in the control group (Figure 3D). Differential abundance analysis further substantiated these findings, identifying three genera of interest (Figure 3C,E). Among these, *Sutterella* was significantly more abundant in the group that consumed disliked food (*q* < 0.05). While not surviving strict multiple-testing correction, *Alloprevotella* and *Succinivibrio* also demonstrated trends toward higher abundance in this group (*q* < 0.2).

### 3.5. Gut Microbiome Composition Is Impacted by a Smaller Meal Size

**Figure 4:**
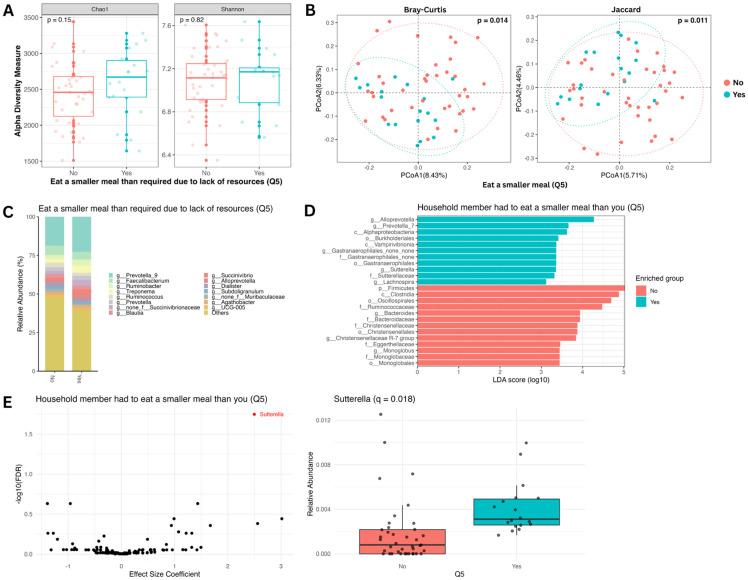
Taxonomic analysis of gut microbiome by question 5 of the HFIAS questionnaire: “Did you or any household member have to eat a smaller meal because there was not enough food (yes: 18 (32%); no: 39 (68%))?” (**A**) Alpha diversity boxplots showing Chao1 (**left**) and Shannon (**right**) indices. Differences in the means of the alpha diversity metrics between yes (blue) and no (red) groups are not significant by the Wilcoxon test. (**B**) Beta diversity PCoA plots showing Bray–Curtis (**left**) and Jaccard (**right**) metrics. Using PERMANOVA, the difference between the beta diversity metric clustering of the two groups is significant (Bray–Curtis *p* < 0.05 and Jaccard *p* < 0.05). (**C**) Relative abundance bar plot showing the top 15 most abundant genera comparing yes and no groups. (**D**) Linear discriminant analysis effect size bar plot. Bars represent taxa with significant group abundance differences (Wilcoxon *p* < 0.01; LDA score > 3). (**E**) Genus-level differential abundance analysis (after adjusting for age, sex, and multiple testing) volcano plot (**left**) and relative abundance boxplot (**right**). The differential abundance of *Sutterella* was found to be significant (*q* < 0.05) between the “yes” and “no” groups.

We also examined whether a reduced meal size due to inadequate food, another indicator of food insecurity, is associated with a change in gut microbiome composition. While no significant differences in alpha diversity were observed (Figure 4A), beta diversity analysis revealed a distinct separation in microbial community structure between individuals who reported eating a smaller meal and those who did not (*q* < 0.02; Figure 4B). LEfSe analysis identified 24 differentially enriched taxa, with 11 associated with those who reported that they ate a smaller meal size and 13 with those who did not (Figure 4D). Notably, differential abundance analysis confirmed a significant increase in the genus *Sutterella* among individuals who reported eating a smaller meal (Figure 4E).

### 3.6. Gut Microbiome Composition and Other Proxy Food Security Variables

We examined the association between additional proxy food security variables from the Household Food Insecurity Access Scale (HFIAS), but these showed no significant associations with microbiome composition. These results are provided in Supplementary Figures S1–S6.

### 3.7. Machine Learning Model Accurately Classifies Food Security Status

**Figure 5:**
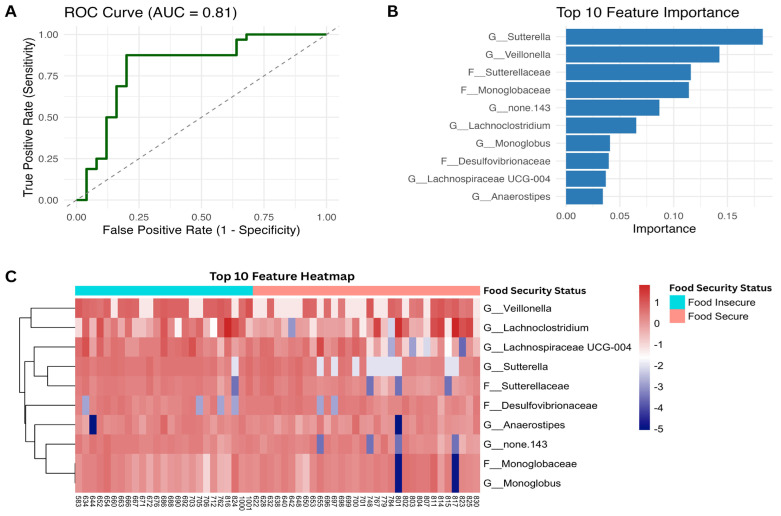
Predictive modeling of food security status using family- and genus-level gut microbiome composition. (**A**) ROC curve displaying the accuracy of a 10-fold cross-validated XGBoost classifier on the study population (*n* = 57). The model achieved a balanced accuracy of 84% and an AUC of 0.81. (**B**) Top 10 most important features bar plot based on the final XGBoost model. (**C**) Heatmap displaying the CLR transform of the top 10 most important features for each participant by food security status.

To evaluate whether gut microbiome composition could predict food security status, we trained a machine learning model using genus- and family-level microbial features. We applied centered log-ratio (CLR) transformation on abundance data and performed 10-fold cross-validation on an XGBoost classifier, which achieved an area under the ROC curve (AUC) of 0.81 (Figure 5A). This indicates gut microbiome composition discriminated food-secure and -insecure individuals accurately. The top 10 most important features identified by the model included genera such as *Sutterella* and *Veillonella* as well as the families *Sutterellaceae* and *Monoglobaceae* (Figure 5B). *Sutterella* emerged as the most predictive taxa (Figure 5B). A heatmap of the top 10 important taxa between groups is visualized in Figure 5C.

## 4. Discussion

In this study, we demonstrated that household food insecurity is associated with a distinct and predictable gut microbiome signature. We found that while alpha diversity did not differ by food security status, beta diversity revealed significant compositional differences in the gut microbiome. We also extended our analysis by examining individual HFIAS questions as specific proxies for dietary deprivation, which revealed significant compositional differences among study subjects who reported consuming a limited variety of foods, disliked foods, and reduced meal size. Notably, differential abundance analyses consistently identified *Sutterella* as significantly more abundant among food-insecure participants. Furthermore, a machine learning model based on genus- and family-level microbial abundance features accurately classified food security status, achieving a balanced accuracy of 84% (AUC = 0.81).

Studies examining the potential association between food insecurity and gut microbiome remain limited and have reported conflicting findings, particularly regarding alpha diversity. While our study and another study in Puerto Rico [30] found no significant association, Mohr et al. [28] reported a significant increase in alpha diversity among food-insecure individuals in U.S. college students. This discrepancy is likely attributable to fundamental differences in study populations and the complex nature of food insecurity itself. Our study enrolled Ethiopian schoolchildren from a similar geographical and cultural setting, where food insecurity is commonly associated with undernutrition [51] and a lack of dietary diversity [52]. In contrast, food insecurity in high-income countries like the USA is often linked to the consumption of energy-dense, nutrient-poor foods [31] and coexists with obesity [4], which may differentially alter the gut microbiome and explain the divergent alpha diversity results. Despite this, our finding of a significant difference in beta diversity by food security status is consistent with that of Mohr et al. [28], suggesting that food insecurity alters microbiome composition, even though its effect on alpha diversity is context-dependent.

In this study, we examined individual HFIAS questions as specific proxies for dietary deprivation in addition to the composite variable food-secure vs food-insecure. We found significant differences in gut microbiome composition, as measured by beta diversity metrics, among study subjects who reported consuming a limited variety of foods, disliked foods, and reduced meal size. To the best of our knowledge, this is the first study to explicitly examine how individual dimensions of food insecurity influence gut microbiome diversity and composition. However, a murine study reported substantial differences in beta diversity but not alpha diversity in pregnant dams and offspring following maternal calorie restriction [53]. These findings, in conjunction with our own observations, suggest that dietary variety, composition, and quality predominantly influence alpha diversity, whereas broader shifts in beta diversity may reflect underlying biological processes associated with malnutrition from food insecurity.

In this study, we observed a significantly higher relative abundance of the genus *Sutterella* among food-insecure individuals compared to food-secure individuals. Additionally, *Sutterella* emerged as the most predictive microbial feature in our machine learning model predicting food security status, and its association with food insecurity is novel. *Sutterella* is a bile-resistant, microaerophilic Gram-negative bacterium that can influence host health through complex immunomodulatory mechanisms, including effects on intestinal inflammation, gut barrier integrity, and the gut–brain axis [54]. Although the exact role of *Sutterella* remains unknown, previous studies have linked elevated levels of *Sutterella* to pro-inflammatory activity [55], involvement in the pathophysiology of neurological and psychological conditions [56], and intestinal infections in humans [57]. Given these diverse health associations, the observed enrichment of *Sutterella* in food-insecure children may reflect the direct impact of dietary patterns commonly associated with food insecurity, such as low fiber intake [58], which can modify the gut environment [59]. Alternatively, elevated *Sutterella* levels may serve as an indirect marker of underlying health conditions in these children, including physiological stress, systemic inflammation [54], or comorbid infections linked to food insecurity, all of which may influence gut microbiome composition. Our cross-sectional design is unable to distinguish the exact role of the higher abundance of *Sutterella* among food-insecure children. A more controlled, mechanistic, and longitudinal study is needed to elucidate the role of *Sutterella* abundance in food-insecure children and assess potential biomarkers.

Our findings should be interpreted with several limitations in mind. First, household food insecurity was assessed using a standardized nine-item experience-based instrument. However, no single universally accepted approach exists for measuring food insecurity. Many organizations have developed various assessment tools [1,35,60], and studies have used different scoring systems and classification thresholds [28,61], which may influence the categorization of food insecurity status and complicate direct comparisons across populations and settings. Differences in instruments and cutoff definitions may therefore affect cross-study interpretation, particularly across diverse geographic and socioeconomic contexts. Second, the food security assessment was based on self-reported data; this may introduce misclassification and recall bias. Although dietary frequency data were collected, these variables were not incorporated into the current microbiome analyses. In addition, detailed quantitative dietary intake measures (e.g., fiber intake or macronutrient composition) were not available. Consequently, we were unable to directly distinguish the relative contributions of dietary quality versus food quantity to the observed differences in the gut microbiome.

Third, while our analysis identifies associations between food insecurity and the gut microbiome, the cross-sectional design limits our ability to draw causal inferences. Furthermore, our study population is not representative of all demographic groups, limiting the generalizability of our findings to populations with different demographics, environmental factors, and dietary behaviors. Future adequately powered, larger independent cohort studies will be necessary to confirm and extend these findings. Although the XGBoost classifier demonstrated good performance (balanced accuracy = 84%; AUC = 0.81), the modest sample size (*n* = 57) and lack of external validation warrant cautious interpretation, and the model should be considered proof-of-concept.

## 5. Conclusions

In summary, our study demonstrates that household food insecurity among Ethiopian schoolchildren is associated with a distinct and identifiable gut microbiome signature. We observed significant differences in microbial composition among participants who reported a limited variety of foods, disliked certain foods, and ate smaller meal portions, compared with their food-secure counterparts. Notably, the consistent enrichment of the genus *Sutterella* across analyses highlights a reproducible microbial feature associated with food insecurity in the study population. Future longitudinal and mechanistic studies will be essential to determine the functional implications of these microbial alterations and to clarify their potential role in mediating the health consequences of food insecurity. By identifying microbiome-associated signatures of food insecurity, our findings provide a foundation for future research exploring whether nutritional or microbiome-informed strategies could mitigate the long-term health effects of food insecurity in vulnerable populations.

## Figures and Tables

**Table 1:** Sociodemographic characteristics of food-secure and food-insecure subjects.

Variable	Food-Secure N = 32 ^1^	Food-Insecure N = 25 ^1^	*p*-Value ^2^
Age (years) mean ± (SD)	10.78 ± 2.41	10.80 ± 1.85	0.9
Weight (kg)	32 ± 9	33 ± 9	0.4
Height (cm)	135 ± 15	139 ± 13	0.3
Sex			0.3
Male	10 (31%)	11 (44%)	
Female	22 (69%)	14 (56%)	
Residence			0.6
Urban	3 (9.4%)	1 (4.0%)	
Rural	29 (91%)	24 (96%)	
Father’s Occupation			>0.9
Farmer	10 (31%)	9 (36%)	
Private business	12 (38%)	8 (32%)	
Daily laborer	3 (9.4%)	2 (8.0%)	
Gov’t employee	7 (22%)	5 (20%)	
Other	0 (0%)	1 (4.0%)	
Wealth Index			>0.9
Wealthy	4 (13%)	2 (8.0%)	
Medium	5 (16%)	4 (16%)	
Poor	23 (72%)	19 (76%)	
Family Size			0.4
≤5	24 (75%)	21 (84%)	
>5	8 (25%)	4 (16%)	
Delivery Mode			0.10
Vaginal delivery	31 (97%)	20 (80%)	
*C*-section	1 (3.1%)	4 (16%)	
Other	0 (0%)	1 (4.0%)	
Feeding Type			0.6
Formula milk	1 (3.1%)	2 (8.0%)	
Breast milk	31 (97%)	23 (92%)	
STH Infection			>0.9
No	15 (47%)	12 (48%)	
Yes	17 (53%)	13 (52%)	

^1^ Mean ± SD; *n* (%). ^2^ Wilcoxon rank sum test; Pearson’s chi-squared test; Fisher’s exact test.

## Data Availability

The processed data supporting the findings of this study, along with the analysis scripts, are publicly available at the GitHub repository https://github.com/azh-a/HFI_gutMicrobiome (accessed on 16 February 2026).

## Supplementary Materials

The following supporting information can be downloaded at: https://www.mdpi.com/article/10.3390/nu18040680/s1, Figure S1–S7.
