# Supplementary material for: Household Food Insecurity Alters Gut Microbiome Composition and Enriches Sutterella in Ethiopian Schoolchildren
Source: Nutrients. 2026 Feb 20;18(4):680. doi: 10.3390/nu18040680 (PMC12942886; doi:10.3390/nu18040680)
Supplement: Supplementary file 1 [file nutrients-18-00680-s001.zip › nutrients-4124265-supplementary.pdf]

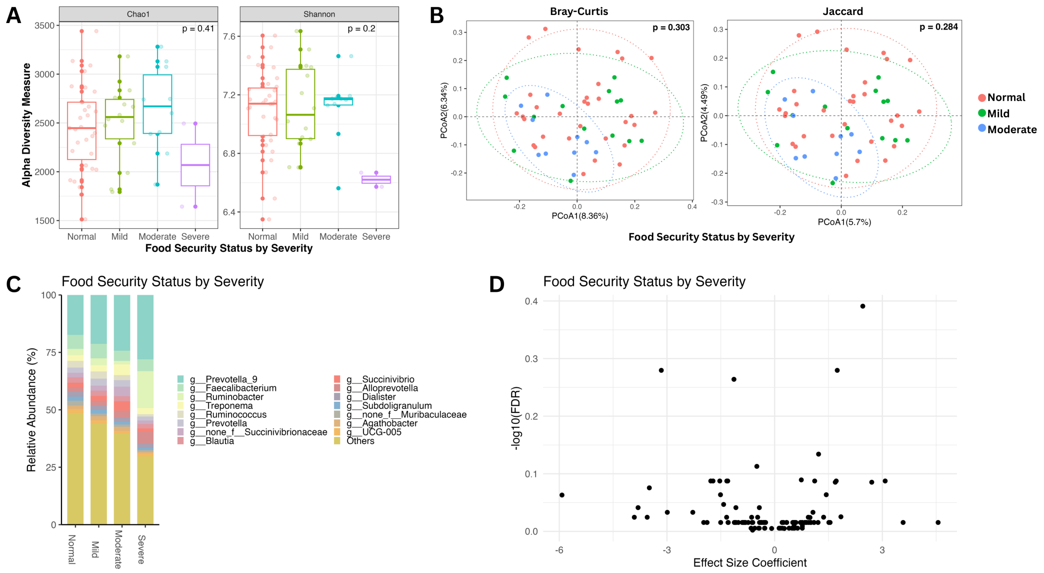

Figure S1: Taxonomic analysis of gut microbiome by food security status severity (A) Alpha diversity boxplots showing Chao1 (left) and Shannon (right) indices. Differences in the means of the alpha diversity metrics between the study groups are not significant by the Kruskal-Wallis test. (B) Beta diversity PCoA plots showing Bray-Curtis (left) and Jaccard (right) metrics. Using PERMANOVA, the difference between the beta diversity metrics centroids of the study groups is not significant. (C) Relative abundance barplot showing top 15 most abundant genera comparing the study groups. (D) Genera level differential abundance analysis (after adjusting for age, sex, and multiple testing) volcano plot. No genera were found to be significant after adjusting for multiple testing.

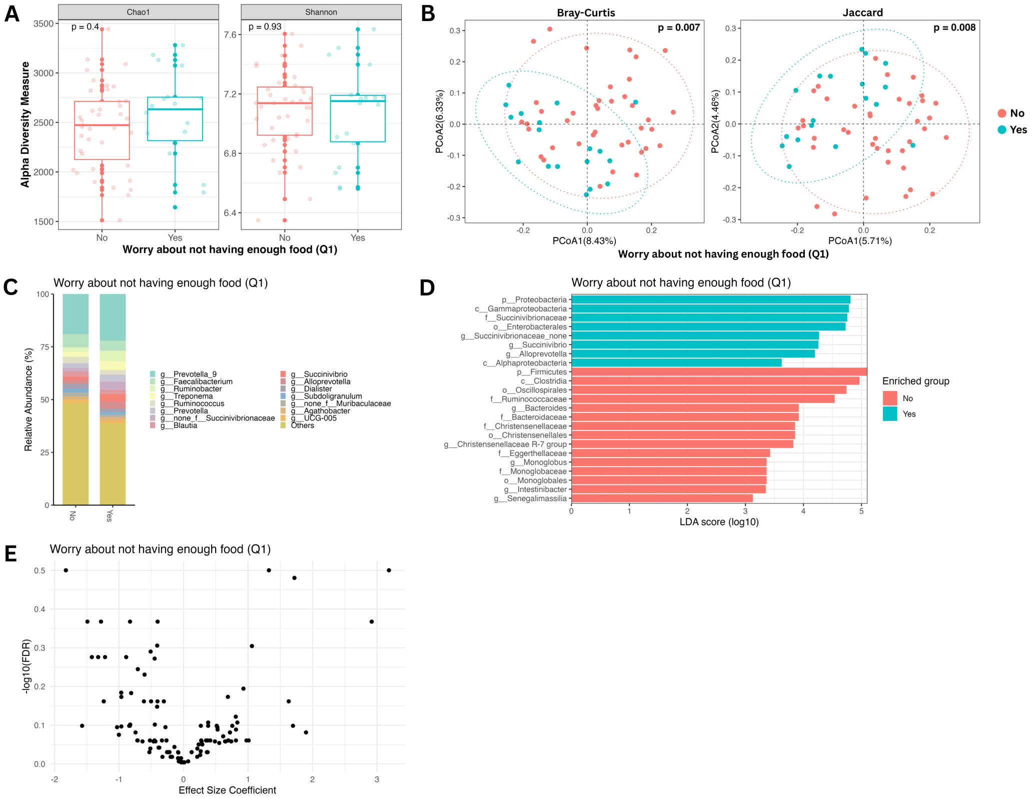

Figure S2: Taxonomic analysis of gut microbiome by question 1 of the HFIAS ques-tionnaire: Did you worry that your household would not have enough food? (A) Alpha diversity boxplots showing Chao1 (left) and Shannon (right) indices. Differences in the means of the alpha diversity metrics between yes (blue) and no (red) groups are not sig-nificant by the Kruskal-Wallis test. (B) Beta diversity PCoA plots showing Bray-Curtis (left) and Jaccard (right) metrics. Using PERMANOVA, the difference between the beta diversity metrics centroids of the two groups is very significant (Bray-Curtis $p < 0.01$ and Jaccard $p < 0.01$). (C) Relative abundance barplot showing top 15 most abundant genera comparing yes and no groups. (D) Linear discriminant analysis effect size barplot. Bars represent taxa with significant group abundance differences (wilcoxon $p < 0.01$, LDA score >3) E) General level differential abundance analysis (after adjusting for age, sex, and multiple testing) volcano plot. No genera were found to be significant after adjusting for multiple testing.

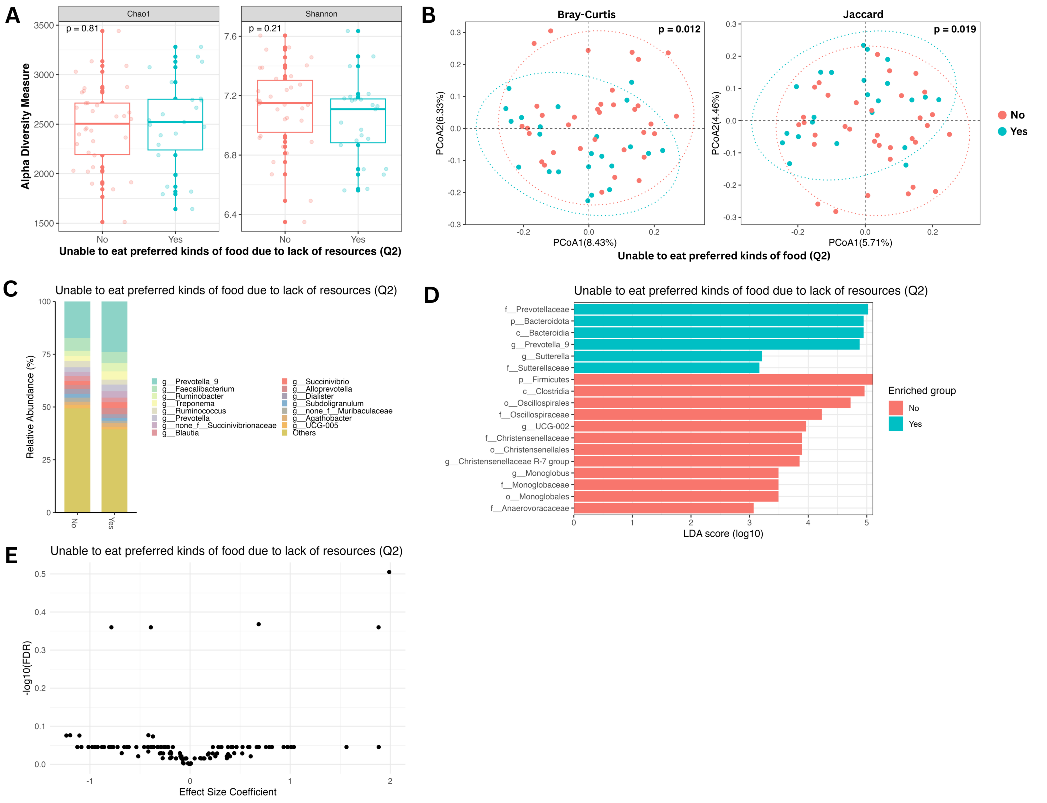

Figure S3: Taxonomic analysis of gut microbiome by question 2 of the HFIAS question-naire: Were you or any household member not able to eat the kinds of foods you preferred because of a lack of resources? (A) Alpha diversity boxplots showing Chao1 (left) and Shannon (right) indices. Differences in the means of the alpha diversity metrics between yes (blue) and no (red) groups are not significant by the wilcoxon. (B) Beta diversity PCoA plots showing Bray-Curtis (left) and Jaccard (right) metrics. Using PER-MANOVA, the difference between the beta diversity metrics centroids of the two groups is significant (Bray-Curtis $p < 0.05$ and Jaccard $p < 0.05$). (C) Relative abundance barplot showing top 15 most abundant genera comparing yes and no groups. (D) Linear discrim-inant analysis effect size barplot. Bars represent taxa with significant group abundance differences (wilcoxon $p < 0.01$, LDA score > 3). (E) Genera level differential abundance analysis (after adjusting for age, sex, and multiple testing) volcano plot. No genera were found to be significant after adjusting for multiple testing.

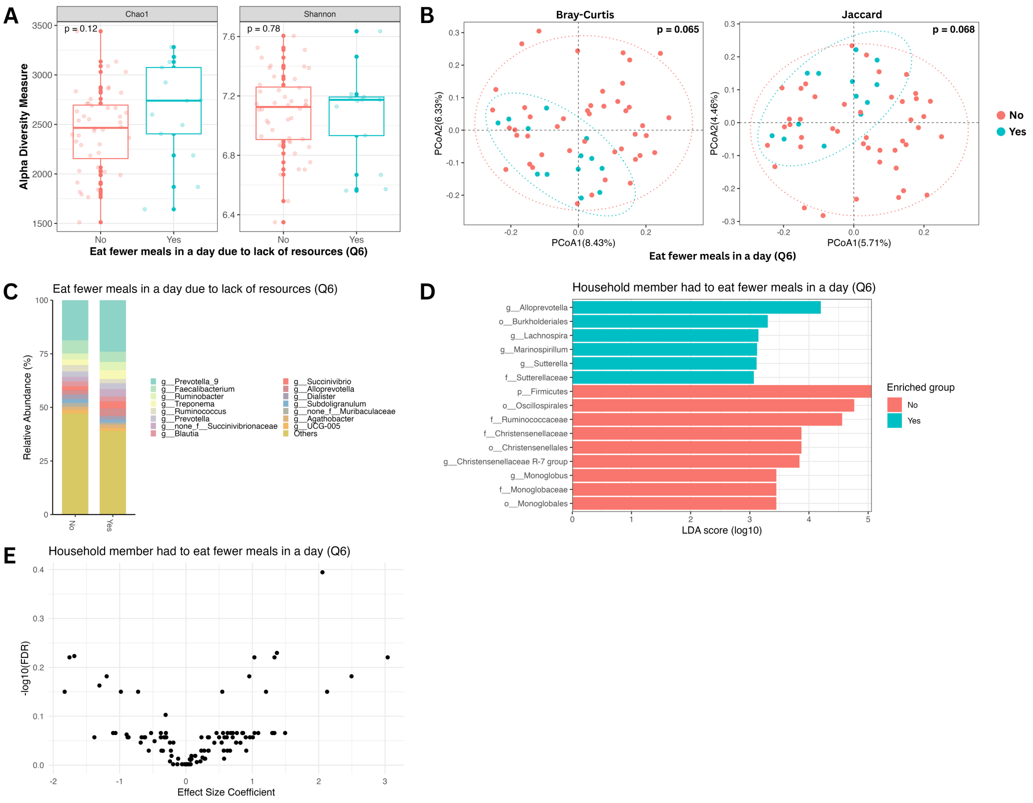

Figure S4: Taxonomic analysis of gut microbiome by question 6 of the HFIAS question-naire: Did you or any household member have to eat fewer meals in a day because there was not enough food? (A) Alpha diversity boxplots showing Chao1 (left) and Shannon (right) indices. Differences in the means of the alpha diversity metrics between yes (blue) and no (red) groups are not significant by the wilcoxon. (B) Beta diversity PCoA plots showing Bray-Curtis (left) and Jaccard (right) metrics. Using PERMANOVA, the difference between the beta diversity metrics centroids of the two groups is moderately significant (Bray-Curtis $p < 0.1$ and Jaccard $p < 0.1$). (C) Relative abundance barplot showing top 15 most abundant genera comparing yes and no groups. (D) Linear discrim-inant analysis effect size barplot. Bars represent taxa with significant group abundance differences (wilcoxon $p < 0.01$, LDA score > 3). (E) Genera level differential abundance analysis (after adjusting for age, sex, and multiple testing) volcano plot. No genera were found to be significant after adjusting for multiple testing.

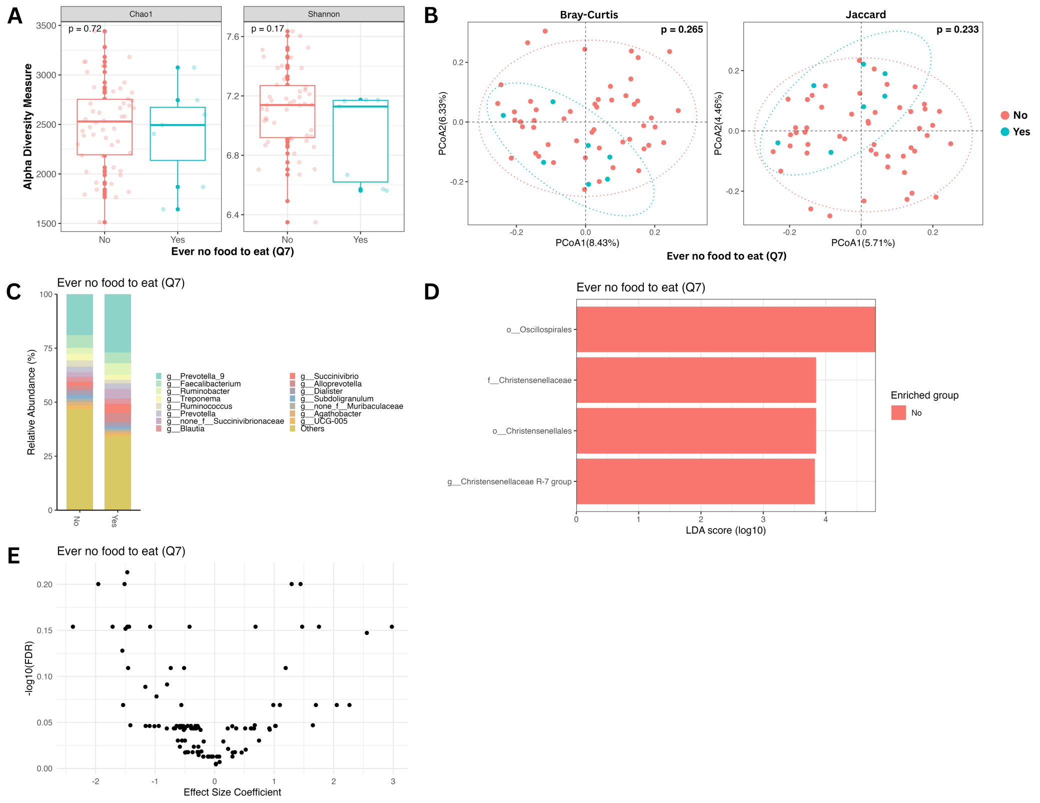

Figure S5: Taxonomic analysis of gut microbiome by question 7 of the HFIAS question-naire: Was there ever no food to eat of any kind in your household because of lack of resources? (A) Alpha diversity boxplots showing Chao1 (left) and Shannon (right) in-dices. Differences in the means of the alpha diversity metrics between yes (blue) and no (red) groups are not significant by the wilcoxon. (B) Beta diversity PCoA plots showing Bray-Curtis (left) and Jaccard (right) metrics. Using PERMANOVA, the difference between the beta diversity metrics centroids of the two groups is not significant.(C) Relative abundance barplot showing top 15 most abundant genera comparing yes and no groups. (D) Linear discriminant analysis effect size barplot. Bars represent taxa with significant group abundance differences (wilcoxon $p < 0.01$, LDA score > 3). (E) Genera level differential abundance analysis (after adjusting for age, sex, and multiple testing) volcano plot. No genera were found to be significant after adjusting for multiple testing.

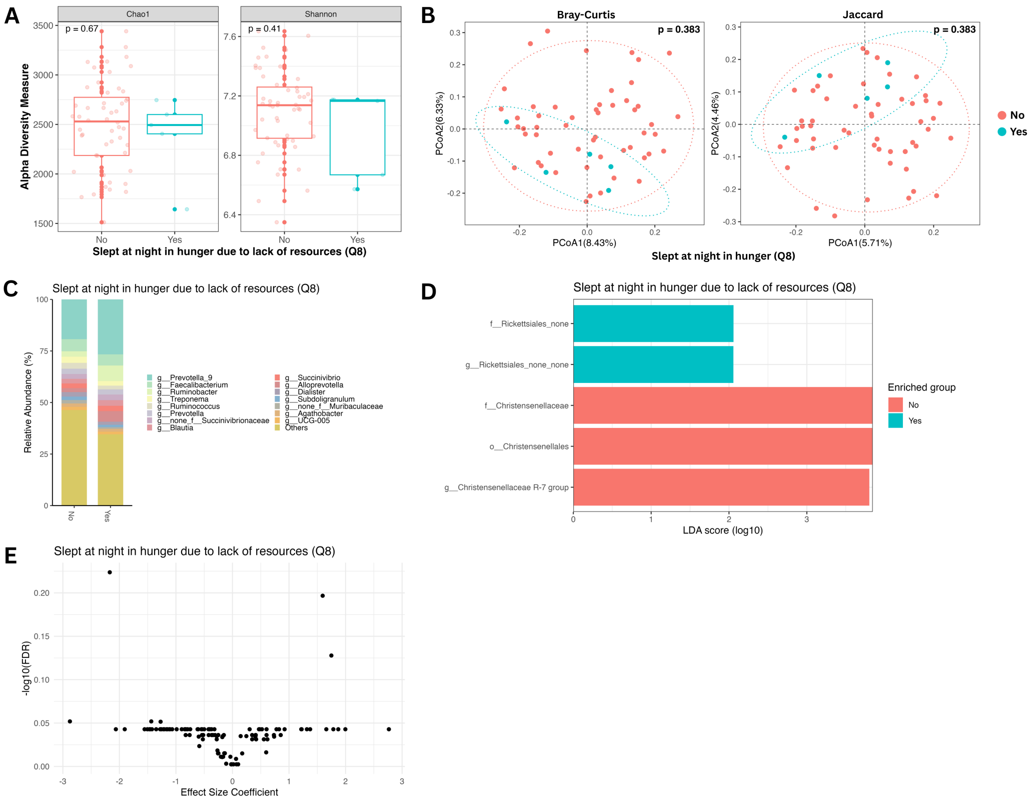

Figure S6: Taxonomic analysis of gut microbiome by question 8 of the HFIAS question-naire: Did you or any household member go to sleep at night hungry because there was not enough food? (A) Alpha diversity boxplots showing Chao1 (left) and Shannon (right) indices. Differences in the means of the alpha diversity metrics between yes (blue) and no (red) groups are not significant by the wilcoxon test. (B) Beta diversity PCoA plots showing Bray-Curtis (left) and Jaccard (right) metrics. Using PERMANOVA, the difference between the beta diversity metrics centroids of the two groups not significant.(C) Relative abundance barplot showing top 15 most abundant genera comparing yes and no groups. (D) Linear discriminant analysis effect size barplot. Bars represent taxa with significant group abundance differences (wilcoxon $p < 0.05$, LDA score > 2). (E) Genera level differential abundance analysis (after adjusting for age, sex, and multiple testing) volcano plot. No genera were found to be significant after adjusting for multiple testing.

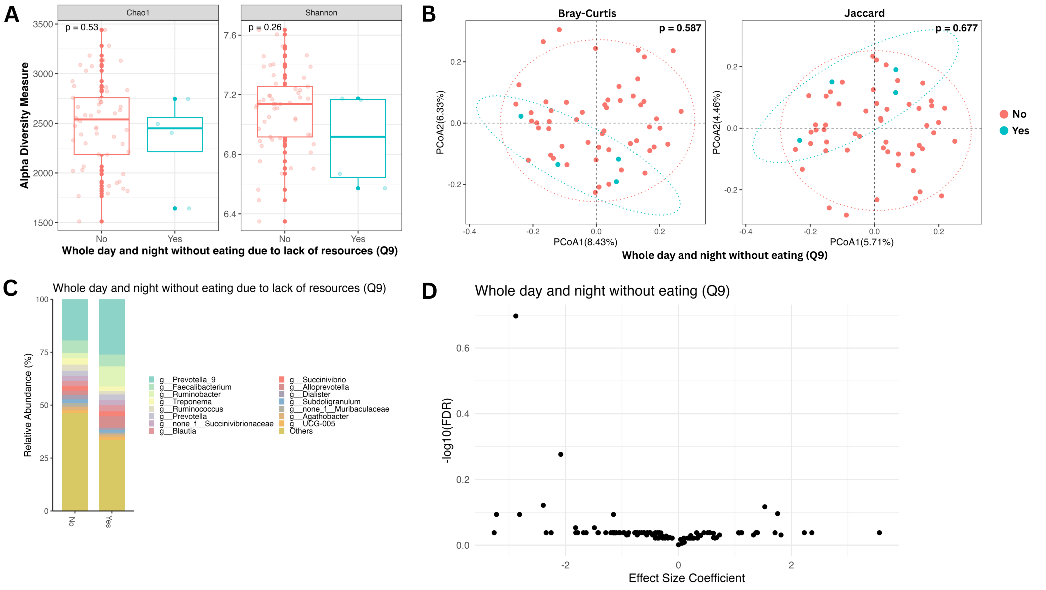

Figure S7: Taxonomic analysis of gut microbiome by question 9 of the HFIAS question-naire: Did you or any household member go a whole day and night without eating anything because there was not enough food? (A) Alpha diversity boxplots showing Chao1 (left) and Shannon (right) indices. Differences in the means of the alpha diversity metrics be-tween yes (blue) and no (red) groups are not significant by the Kruskal-Wallis test. (B) Beta diversity PCoA plots showing Bray-Curtis (left) and Jaccard (right) metrics. Us-ing PERMANOVA, the difference between the beta diversity metrics centroids of the two groups is not significant. (C) Relative abundance barplot showing top 15 most abundant genera comparing yes and no groups. (D) Genera level differential abundance analysis (after adjusting for age, sex, and multiple testing) volcano plot. No genera were found to be significant after adjusting for multiple testing.
